# Fibromuscular dysplasia in an accessory renal artery causing renovascular hypertension: a case report

**DOI:** 10.1186/1752-1947-1-58

**Published:** 2007-07-31

**Authors:** Abdel-Rauf Zeina, Wolfson Vladimir, Elisha Barmeir

**Affiliations:** 1 Department of Radiology & MAR Imaging Institute, Bnai-Zion Medical Center, Faculty of Medicine, Technion, Haifa, Israel

## Abstract

**Background:**

Renovascular hypertension is defined as hypertension caused by renal artery stenosis. The two main etiologies are atherosclerosis and fibromuscular dysplasia. Fibromuscular dysplasia in an accessory renal artery as a cause of renovascular hypertension is uncommon.

**Case presentation:**

In this report, we present a relatively uncommon case of renovascular hypertension in a 35-year-old female with a history of intractable hypertension as a result of fibromuscular dysplasia involving an accessory renal artery. Selective renal angiography was performed and revealed a single renal artery on the right and two renal arteries supplying the left kidney, upper and lower poles. Selective renal angiography showed the typical fibromuscular dysplasia lesion characterized by its classic "string of beads" appearance, consisting of alternating areas of narrowing and dilatation, located in the middle portion of the lower left renal artery (accessory artery) associated with moderate stenosis. Percutaneous balloon dilatation of the stenotic lesion was successfully performed. Following angioplasty, her blood pressure normalized over a period of several months using a single antihypertensive medication (rather than 3 medications).

**Conclusion:**

Fibromuscular dysplasia in an accessory renal artery can, even though rarely, be responsible for renovascular hypertension. Selective renal angiography is the 'gold standard' test and should be performed when renovascular intervention is contemplated.

## Background

Renovascular hypertension (RVH) is defined as hypertension caused by renal artery stenosis (RAS) and accounts for less than 5% of all cases of hypertension in the general population [1]. The two main etiologies of RAS are atherosclerosis and fibromuscular dysplasia (FMD). Atherosclerosis accounts for 70–90% of cases of RAS and usually involves the ostium and proximal portion of the main renal artery [2]. FMD is a non-atherosclerotic, non-inflammatory vascular disease, responsible for 10–30% of cases of RAS [2,3]. FMD may involve any layer of a visceral artery, and it may be classified as intimal, medial, or adventitial. The medial form may result in arterial stenosis causing organ ischemia or infarction. Other rare causes of RAS are Takayasu's arteritis, radiation-induced arteritis, spontaneous dissecting aneurysm and Von Recklinghausen's disease.

Selective renal angiography (SRA) remains the gold standard for the diagnosis of renal artery stenosis. However, noninvasive diagnostic techniques such as Doppler ultrasound (DU), MR angiography (MRA) and CT angiography (CTA) have proved to be accurate in assessment of RAS and provide valuable alternatives to diagnostic angiography [4-6]. In this paper, we present a case of FMD involving an accessory renal artery causing intractable hypertension diagnosed by SRA. We also discuss and illustrate the angiographic appearance of FMD, essential in making the correct diagnosis and planning patient treatment.

## Case presentation

**Figure 1:**
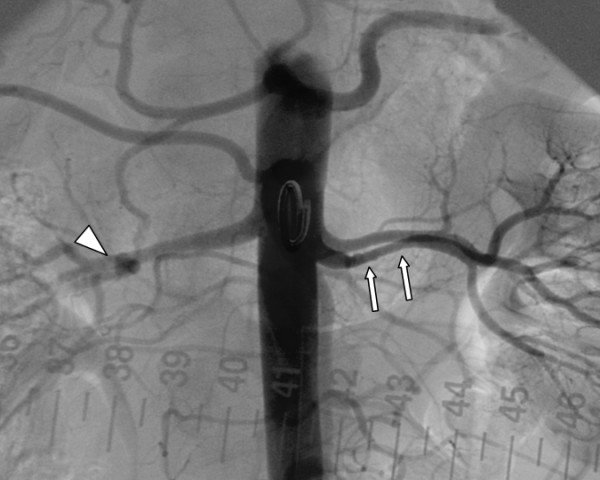
Renal artery angiography in a 35-year-old woman with unexplained hypertension showing the typical "string-of-beads" sign (arrows) characteristic for FMD involving the lower left renal artery (accessory artery). The arrowhead indicates a small saccular aneurysm at the distal portion of right renal artery.

**Figure 2:**
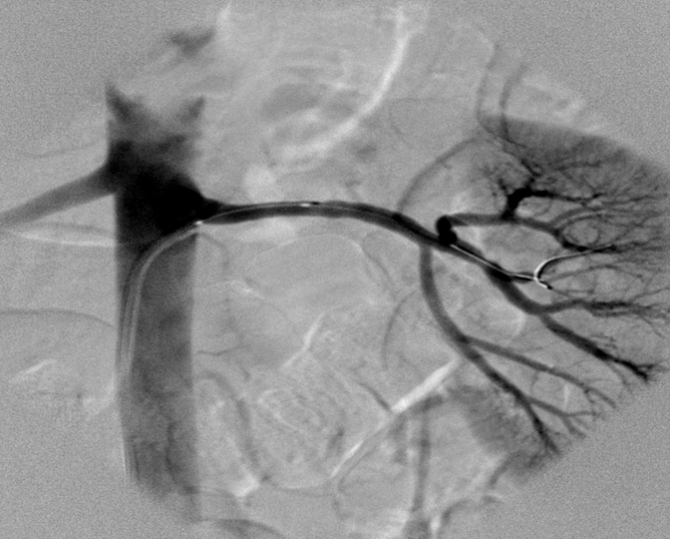
Selective renal angiography (left lower renal artery) after successful percutaneous balloon dilatation of the stenotic lesion.

A 35-year-old female with a history of intractable hypertension (for the duration of a year), probably renovascular, was referred by her nephrologist to our department for SRA. She was a smoker. The patient denied any family history of hypertension. Her physical examination revealed a blood pressure of 150/100 mmHg (multiple readings taken from both arms on different occasions were similar). Her cardiovascular, respiratory, and central nervous system examinations were unremarkable. No evidence of retinopathy on fundus examination. There was no carotid, abdominal or femoral arterial bruits. ECG, chest radiograph and transthoracic echocardiography were normal. Her blood urea nitrogen (BUN) and serum creatine were within normal limits. The patient received 3 antihypertensive medications including a beta-blocker, diuretic and a calcium channel blocker. SRA was performed and revealed a single renal artery on the right and two renal arteries supplying the left kidney, upper and lower poles (anatomical variation). SRA showed the typical FMD lesion which is characterized by its classic "string of beads" appearance, consisting of alternating areas of narrowing and dilatation, located in the middle portion of the lower left renal artery (accessory artery) associated with moderate stenosis (reduction in luminal diameter greater than 50%) (Figure 1). The upper left renal artery was preserved. In addition, SRA revealed a small saccular aneurysm of the distal right renal artery (Figure 1). Percutaneous balloon dilatation of the stenotic lesion (middle portion of the accessory renal artery) was successfully performed (Figure 2). Following angioplasty, her blood pressure normalized over a period of several months using a single antihypertensive medication (atenolol 50 mg once daily), rather than 3 medications.

## Discussion

FMD is a nonatherosclerotic angiopathy of unknown etiology. Medial FMD represents the most common type and is characterized by the classic "string of beads" appearance. FMD usually affects females between 15 and 50 years of age, frequently involves the mid or/and distal segments of the renal artery and is bilateral in 2/3 of the patients [7]. It is the most common cause of RVH in children. Renal artery stenosis secondary to FMD may affect pregnant women and thus remains an important consideration as a cause of secondary hypertension during pregnancy. Thorsteinsdottir et al. [8] have reported on a series of patients with poor pregnancy outcomes due to severe preeclampsia in patients with RAS. They also showed that some of these women had successful pregnancies after revascularization.

RVH is the clinical consequence of renin-angiotensin-aldosterone system activation as a result of renal ischemia. Unilateral renal ischemia initiates an increased secretion of renin, which accelerates the conversion of angiotensin I to angiotensin II and enhances the adrenal release of aldosterone. The result is profound angiotensin-mediated vasoconstriction and aldosterone-induced sodium and water retention, causing RVH. Goldblatt [9] (1934) demonstrated that occlusion of the renal artery causes ischemia, which then causes an elevation of blood pressure by triggering the release of renin. In the 2-kidney 1-clip model of Goldblatt, an obstruction is produced in one renal artery by a mechanical clip while the contralateral kidney is functioning and left unobstructed. The clip causes renal ischemia and consequently increased renin secretion from the stenotic kidney. Nephrectomy of ischemic kidney will cure hypertension. In the setting of a solitary kidney (1-kidney 1-clip model) and in the 2-kidney 2-clip model (clips obstruct both renal arteries) there is no functioning contralateral kidney that can excrete the overload of water and sodium.

In about one-third of the general population there are variations in number, location, and branching patterns of the renal arteries, with over 30% of subjects having one or more accessory renal arteries [10]. This is clinically important because RAS in an accessory renal artery can, even though rarely, be responsible for RVH. In our patient, despite the preserved left upper renal artery supplying the upper pole of the left kidney, RVH developed. Lesions occluding more than 50% of the diameter of the artery are considered significant. Though there are no clear-cut indications for intervention, the following criteria may be used as a guide for renal artery revascularization: recent onset of hypertension in whom the goal is to cure the hypertension, drug-refractory hypertension (three or more drugs), intolerance to antihypertensive medications, progressive renal insufficiency/failure and finally episodes of flash pulmonary edema. Clinical response in patient with RVH consists of a decrease in serum creatinine level of 30 μmol/l or a reduction in the number of medications required for blood pressure control after renal artery angioplasty or surgery [11]. Following angioplasty, the blood pressure in our patient returned to normal on a single antihypertensive medication (rather than 3 medications before the procedure). Renal artery aneurysm, as reported in our patient involving the right renal artery, is considered a complication of FMD and does not represent distinct histopathological categories. Renal artery dissection may also complicate FMD.

SRA remains the gold standard for the diagnosis of renal artery stenosis. However, because of the invasive nature of this procedure, various non-invasive imaging modalities have been applied to detect renal artery stenosis including DU, MRI and CTA. Duplex ultrasonography can provide images of the renal arteries and asses blood-flow velocity and pressure waveforms, however there is a 10% to 20% rate of failure due to the operator's inexperience, the presence of obesity or bowel gas, respiratory renal movements, and poor patient compliance. In addition, visualization of a single normal renal artery does not exclude the possibility of a stenotic accessory renal artery. At present the most important role of ultrasonography is its apparent ability to predict functional recovery based on the measurement of resistive index. Captopril renography is a non-invasive and safe technique to evaluate renal blood flow and excretory function providing indirect evidence of the presence of renal artery stenosis and has proven helpful in screening patients with this condition. The efficacy of the test is increased when 25–50 mg of captopril is administered one hour prior to the injection of the radioisotope. However, data concerning the reliability of this technique are inconsistent and vary among studies. The sensitivity and specificity of captopril renography decrease in the presence of azotemia, bilateral disease, or disease in a solitary functioning kidney [12].

Multidetector CTA is the most widely used scan in the diagnosis of RAS. It permits rapid volumetric acquisition with high-contrast enhancement of the vessel lumen. Due to the high spatial resolution (submillimeter) it provides excellent visualization of the renal arteries as well as side branches. The study conducted by Sabharwal et al [13], reported a 100% diagnostic accuracy of CTA in the detection of renal FMD (of either main renal or accessory arteries). Similar results have been reported by others [14,15]. Advantages of CTA over CA include non-invasiveness, time and cost efficiency, low complication rate profile, demonstration of extraluminal anatomical structures such as the renal parenchyma and the visualization of not only the arterial lumen but also the arterial wall. MR angiography, in view of the absence of ionizing radiation and the possibility of using non nephrotoxic contrast medium, is widely accepted for the detection of RAS. Nonetheless, this modality has the disadvantage of relatively low spatial resolution to depict segmental stenosis in distal, intrarenal and accessory renal arteries which are better evaluated by CTA. Investigators in a Dutch multicenter trial of this method reported sensitivities and specificities for the grading of atherosclerotic renal artery stenosis of less than 80%. Sensitivity decreased from 78% to 22% when only patients with FMD were included in the study population [10].

## Conclusion

Fibromuscular dysplasia in an accessory renal artery can, even though infrequently, be responsible for renovascular hypertension. Selective renal angiography is the 'gold standard' test and should be performed when renovascular intervention is contemplated.

## Abbreviations

CTA; computed tomography angiography, FMD; fibromuscular dysplasia, MDCT; multidetector computed tomography, RAS; renal artery stenosis, RVH; Renovascular hypertension.

## Competing interests

The author(s) declare that they have no competing interests.

## Authors' contributions

All authors have read and approved the final manuscript.

ARZ (Consultant Radiologist): Involved in the conception of the report, literature review, manuscript preparation, manuscript editing and manuscript submission.

WV (Consultant Radiologist): Involved in the manuscript editing and manuscript review.

EB (Consultant Radiologist): Involved in the manuscript editing and manuscript review.
